# Molecular Identification, Virulence Factors, and Antifungal Susceptibility Profiles of *Candida* Isolates from Clinical Samples of Intensive Care Patients

**DOI:** 10.3390/antibiotics15020197

**Published:** 2026-02-10

**Authors:** Zeynep Çelik, İbrahim Halil Kılıç, Semih Tokak, Fatma Esenkaya Taşbent

**Affiliations:** 1 Department of Biology, Faculty of Arts and Sciences, Gaziantep University, Gaziantep 27310, Türkiye; zc211002@mail2.gantep.edu.tr; 2 Department of Medical Microbiology, Faculty of Medicine, Konya Chamber of Commerce Karatay University, Konya 42020, Türkiye; semih.tokak@karatay.edu.tr; 3 Department of Medical Microbiology, Faculty of Medicine, Necmettin Erbakan University, Konya 42090, Türkiye; ftasbent@erbakan.edu.tr

**Keywords:** *Candida*, ITS-sequence, MALDI-TOF MS, CHROMagar, virulence factors, antifungal susceptibility

## Abstract

**Background/Objectives:**  *Candida* infections constitute a significant category of healthcare-associated infections. In studies aiming to develop new antifungal agents against *Candida* species, the importance of their virulence factors has been emphasized. **Methods:** This study included 100 *Candida* isolates obtained from patients hospitalized in intensive care units. Standard microbiological and molecular methods were employed for species identification. Virulence factors were determined through protease, phospholipase, hemolysis, and biofilm activity assays per-formed on the *Candida* strains. The EUCAST liquid microdilution method was used to assess antifungal susceptibility. **Results:** Based on sequencing results, 39 isolates were identified as *Candida albicans* and 61 as non-*albicans Candida* species. The accuracy of species identification was found to be 71% for Chromagar *Candida* and 87% for the MALDI-TOF MS system, compared to sequencing. Protease activity was positive in 52% of the isolates, phospholipase in 42%, hemolytic activity in 77%, and biofilm formation in 48%. Kruskal–Wallis analysis revealed no statistically significant interspecies differences in MIC distributions for amphotericin B, fluconazole, itraconazole, or nystatin (*p* > 0.05), although species-specific trends were observed, with higher fluconazole MICs in *C. albicans* and lower MIC values in *C. tropicalis.*  **Conclusions:** Determining the distribution of *Candida* species, as well as their virulence factors and antifungal MIC profiles, is of great importance for developing appropriate treatment strategies and reducing related morbidity and mortality.

## 1. Introduction

*Candida* infections, like many infectious diseases, have undergone significant epidemiological and clinical changes in recent years, yet they continue to be the most commonly observed invasive fungal infection among patients hospitalized in tertiary care hospitals [1]. Candidiasis encompasses a range of infections from skin or mucosal infections to bloodstream infection (candidemia), endocarditis, central nervous system infection, urinary tract candidiasis, and chronic disseminated candidiasis [2]. Invasive candidiasis is particularly associated with high morbidity and mortality in immunocompromised and critically ill patients [3].

Isolation rates vary, but *C. albicans* remains the most common etiological agent, followed by *C. glabrata*, *C. tropicalis*, *C. parapsilosis*, and *C. krusei*. These species have comprised over 95% of candidiasis cases in the past thirty years [4]. The incidence of invasive infections caused by *Candida* spp. has increased in recent years, including a rise in the proportion of infections caused by non-*albicans Candida* (NAC) species [5]. This increase has been associated with factors such as overuse of broad-spectrum antibiotics and antifungals, chemotherapy, organ transplantation, long-term catheter use, and inadequate performance of routine microbiological tests [6]. In 2012, the annual global incidence of invasive candidiasis was estimated at about 400,000 cases [7]; by 2024, approximately 1.5 million people were estimated to develop invasive candidiasis each year worldwide, with about 1 million deaths (~63.6%) attributable to it [8].

Globally, increased prophylactic and therapeutic use of antifungal drugs for *Candida* infections has led to reduced susceptibility and the emergence of resistant *Candida* species. As a result, many regions see widespread *Candida* infections, creating an increasing need for in vitro antifungal susceptibility testing to guide effective therapy [9,10].

The pathogenicity of *Candida* species occurs via various virulence factors, including adhesion to host cell surfaces, invasion, biofilm formation, the yeast-to-hypha transition, production of hydrolytic enzymes (proteases, phospholipases, hemolysins) that damage host tissues, and evasion of immune cells [11]. The expression of these factors can vary depending on the infecting species, geographic region, site of infection, and the host’s immune status.

In this study, we aimed to identify in detail the species and characteristics of *Candida* strains isolated from clinical specimens of ICU patients. CHROMagar Candida colony morphology, MALDI-TOF MS, and ITS-PCR sequencing were used to compare identification accuracy. Additionally, to assess virulence potential, we evaluated secreted aspartic protease (SAP), phospholipase, hemolytic activity, adhesion, and biofilm formation. The antifungal susceptibility profiles of the isolates were also determined to identify clinically relevant resistance patterns.

## 2. Results

### 2.1. Patient Characteristics and Specimen Distribution

**Figure 1:**
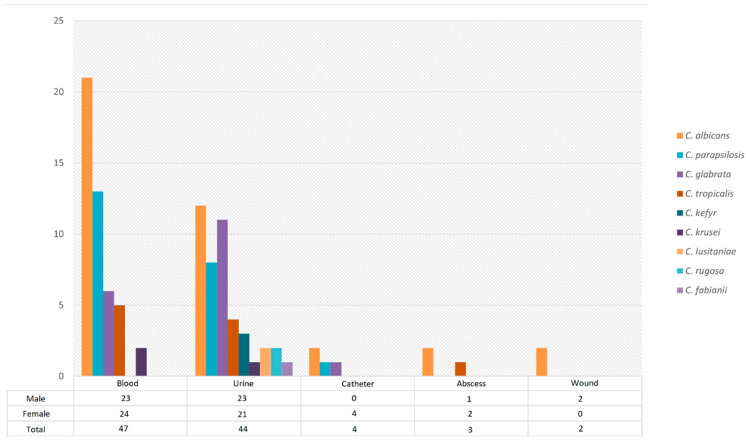
Distribution of Candida isolates by specimen type based on ITS sequence-confirmed molecular identification.

A total of 100 *Candida* isolates were obtained from patients hospitalized in intensive care units. The patients’ ages ranged from 19 to 94 years (mean ± SD: 56 ± 2), with 49 males (49%) and 51 females (51%). The isolates were recovered from blood (47%), urine (44%), catheter tips (4%), abscess samples (3%), and wound swabs (2%) (Figure 1).

### 2.2. Species Identification and Method Comparison

Based on ITS rDNA sequencing, nine *Candida* species were identified among the 100 clinical isolates. The most frequently detected species were *C. albicans* (39%), followed by *C. parapsilosis* (22%), *C. glabrata* (18%), and *C. tropicalis* (10%). Less frequently isolated species included *C. kefyr* (3%), *C. lusitaniae* (3%), *C. krusei* (2%), *C. rugosa* (2%), and *C. fabianii* (1%).

The accuracy of species identification using CHROMagar Candida and MALDI-TOF MS was evaluated by comparison with ITS sequencing results (Table 1). Overall, MALDI-TOF MS demonstrated higher concordance with sequencing than CHROMagar Candida. MALDI-TOF MS correctly identified all isolates of *C. tropicalis*, *C. kefyr*, *C. rugosa*, and *C*. *fabianii*. In contrast, misidentifications were observed mainly among *C. albicans*, *C. parapsilosis*, and *C. glabrata* isolates, although the overall agreement with sequencing remained high.

CHROMagar Candida showed lower specificity, particularly for non-*albicans Candida* (NAC) species. While *C. albicans* and *C. tropicalis* were identified with relatively higher accuracy, several NAC species were misidentified or could not be reliably distinguished based solely on colony color and morphology.

### 2.3. Virulence Factor Profiles

**Figure 2:**
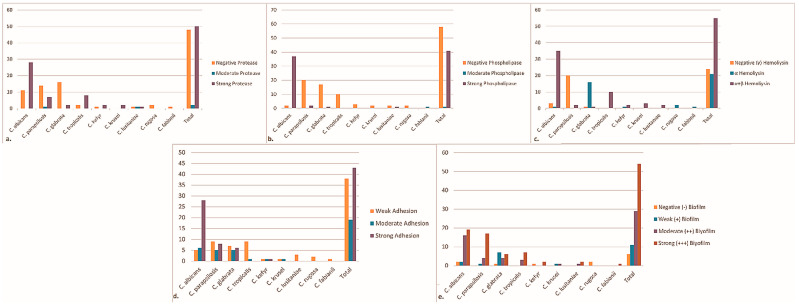
Virulence factor activities of Candida isolates: (**a**) protease; (**b**) phospholipase; (**c**) hemolysis; (**d**) adhesion; (**e**) biofilm.

Protease activity was detected in 52% of all isolates. The highest frequency of protease positivity was observes in *C. albicans*, followed by *C. parapsilosis* and *C. tropicalis* (Figure 2a). No protease activity was detected in *C. rugosa* or *C. fabianii* isolates.

Phospholipase activity was observed in 42% of the isolates. This activity was predominantly associated with *C. albicans*, whereas phospholipase positivity was rare among NAC species and absent in *C. tropicalis*, *C. kefyr*, *C. krusei*, and *C. rugosa* isolates (Figure 2b).

Hemolytic activity was detected in 76% of isolates. Among hemolysis-positive strains, most exhibited a double hemolytic pattern (α + β), while a smaller proportion showed only α-hemolysis. Hemolytic activity was most frequently observed in *C. albicans* and *C. tropicalis*, whereas several NAC species demonstrated weaker or absent hemolytic profiles (Figure 2c).

Adhesion assays revealed that 43% of isolates exhibited strong adhesion, 19% moderate adhesion, and 38% weak adhesion. Strong adhesion was most commonly observed among *C. albicans* isolates (Figure 2d).

Biofilm formation was detected in 48% of the isolates. Among biofilm-positive strains, 68.75% were moderate biofilm producers and 31.25% were strong producers. Strong biofilm formation was most frequently associated with *C. parapsilosis*, followed by *C. glabrata*, while *C. albicans* isolates predominantly exhibited moderate biofilm-forming capacity (Figure 2e).

### 2.4. Statistical Evaluation of Virulence Traits

Nine species were identified from 100 clinical *Candida* isolates; their numbers and statistical significance are shown in Table 2. For statistical analyses, only species represented by at least 10 isolates (*C. albicans*, *C. parapsilosis*, *C. glabrata*, and *C. tropicalis*) were included (Table 2).

**Figure 3:**
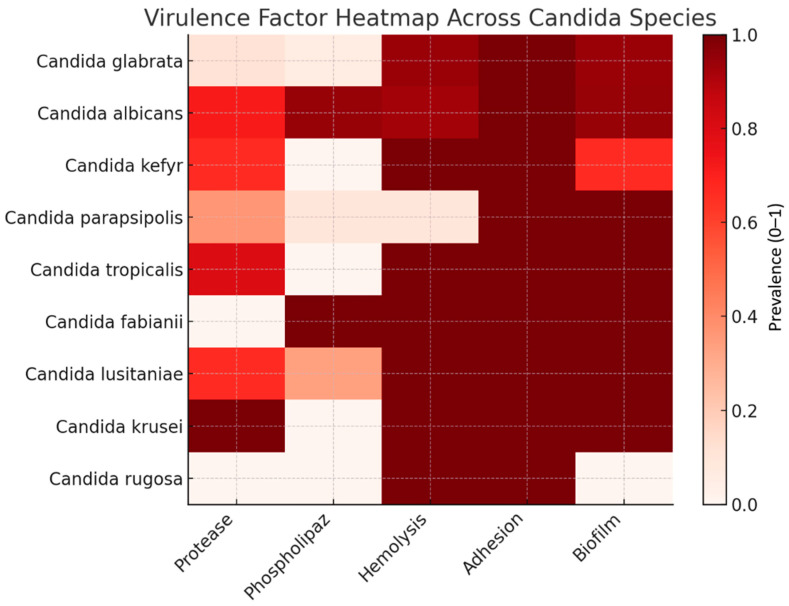
Heatmap showing the prevalence of virulence factors (protease, phospholipase, hemolysis, adhesion, biofilm) among *Candida* species.

The distribution of virulence factors among species was evaluated using the Chi-square (χ^2^) test, and the results are shown in Table 3. Chi-square analysis demonstrated a highly significant association between species and protease activity (*p* < 0.001), as well as phospholipase activity (*p* < 0.0001). Hemolytic activity also differed significantly among species (*p* = 0.011). In contrast, no statistically significant interspecies differences were observed for adhesion or biofilm formation (*p* > 0.05) (Table 3). The relative distribution of virulence traits across species is summarized in Figure 3. High enzymatic activity is mainly observed in *C. albicans* and *C. tropicalis*, whereas *C. glabrata* exhibits a markedly low enzymatic profile. Adhesion and biofilm formation are uniformly prevalent across species.

### 2.5. Antifungal Susceptibility Profiles

Antifungal susceptibility testing was performed for amphotericin B, fluconazole, itraconazole, and nystatin using the EUCAST broth microdilution method. Kruskal–Wallis analysis revealed no statistically significant differences in MIC distributions among the included species for any of the tested antifungal agents (*p* > 0.05) (Table 4).

**Figure 4:**
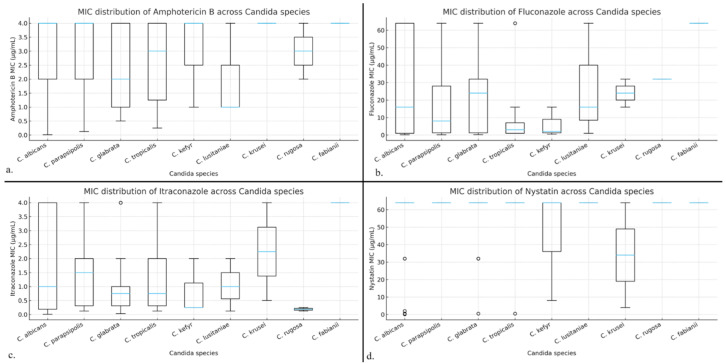
Boxplot representation of (**a**) Amphotericin B; (**b**) Fluconazole; (**c**) Itraconazole and (**d**) nystatin MIC distributions across *Candida* species. No significant interspecies differences were observed (Kruskal–Wallis, *p* > 0.05).

Despite the absence of statistically significant interspecies differences, species-specific trends were observed. *C. albicans* isolates tended to exhibit higher fluconazole MIC values compared to other species, whereas *C. tropicalis* generally showed lower MIC values across all tested antifungals. Variability in itraconazole susceptibility was noted particularly among *C. parapsilosis* and *C. glabrata* isolates (Figure 4).

Correlation analyses using Spearman’s test, a Mann–Whitney U test, and binary logistic regression demonstrated no significant associations between individual virulence factors and antifungal MIC values (*p* > 0.05). Biofilm formation showed a weak positive correlation with fluconazole MIC levels (ρ = 0.21), although this association did not reach statistical significance. Logistic regression analysis indicated a non-significant trend toward increased odds of fluconazole resistance among biofilm-positive isolates. Although these associations did not reach statistical significance, the observed directional trends suggest that biofilm formation may contribute to reduced azole susceptibility at the phenotypic level, warranting further investigation in larger cohorts.

## 3. Discussion

In recent years, the incidence of invasive *Candida* infections has increased substantially, particularly among patients admitted to intensive care units (ICUs). This rise has been attributed to the widespread use of broad-spectrum antibiotics, prolonged hospitalization, invasive medical devices, and immunosuppressive conditions [12]. Although *C. albicans* remains the most frequently isolated species worldwide, a progressive shift toward non-*albicans Candida* (NAC) species has been consistently reported, with important implications for diagnosis, virulence, and antifungal therapy [13].

NAC species, most commonly *C. parapsilosis*, *C. glabrata*, and *C. tropicalis*, are increasingly encountered worldwide [14]. Studies from China and multicentre cohorts have reported that NAC species now equal or exceed *C. albicans* in prevalence, with *C. glabrata*, *C. tropicalis*, and *C. parapsilosis* being the most frequent NAC species [15,16]. Long-term surveillance data demonstrates a declining trend in *C. albicans* isolation accompanied by a steady increase in NAC species over time [17]. Regional studies from India and Brazil further confirm marked geographic variability in species distribution, with high proportions of NAC species [18,19]. Studies from Türkiye consistently report a predominance of NAC species in clinical isolates, particularly *C. parapsilosis* and *C. glabrata*, and recent data also document the emergence of rare species such as *C. fabianii* [20,21,22,23].

The epidemiological distribution of *C. albicans* and NAC species varies significantly depending on geographic location, the healthcare facility involved, and the patient population [24]. In this study, *C. albicans* accounted for 39% of isolates, while NAC species collectively represented 61%. Among NAC species, *C. parapsilosis*, *C. glabrata*, and *C. tropicalis* were the most prevalent. This distribution is consistent with reports from Türkiye [20,21,22,23] and other regions [14,15,16,17,18,19], which have documented a declining proportion of *C. albicans* and a parallel increase in NAC species in ICU settings. The observed species distribution likely reflects local epidemiological patterns, antifungal usage practices, and patient-related risk factors. As this study was conducted at a single tertiary-care center, the findings may not fully capture nationwide variability; however, the data are representative of ICU populations commonly encountered in similar healthcare settings.

In recent years, there has been a clear shift from conventional diagnostic approaches toward proteomic and nucleic acid amplification-based techniques. Nevertheless, traditional diagnostic methods continue to constitute the cornerstone of fungal infection diagnosis due to their ease of use, cost-effectiveness, and long-standing routine application in clinical practice [25,26].

The performance of identification of *Candida* species using chromogenic media is subject to various factors, including the brand of the medium and the site of sample collection [25]. Chromogenic media have been observed to lack the capacity to differentiate some clinically relevant *Candida* species [27]. Furthermore, within the same species, variations in color and morphology have been documented [28,29,30]. Nerurkar and colleagues [31] reported that CHROMagar Candida medium reliably identified *C. albicans* and *C. tropicalis.* Vecchione et al. [32] stated that the differentiation of *C. albicans*, *C. tropicalis*, and *C. krusei* strains was possible on chromogenic media in accordance with the manufacturer’s recommendations. In this study, CHROMagar Candida demonstrated higher identification accuracy for *C. albicans*, *C. tropicalis*, and *C. parapsilosis*, but showed lower performance for *C. glabrata* and *C. krusei*, failed to identify some rare species, and misidentified a *C. krusei* isolate with intrinsic fluconazole resistance as *C. glabrata*.

The use of automated devices such as MALDI-TOF MS has been shown to reduce identification time, hospital stay duration, and overall costs com-pared to conventional methods [33]. A recent study reported that when rDNA sequencing was used as the reference method, the correct identification rates for MALDI-TOF MS and conventional methods were 98.3% and 96.5%, respectively [34,35]. In 2021, Meena and colleagues demonstrated that MALDI-TOF MS successfully identified all tested *Candida* isolates and confirmed it as a rapid, reliable, and cost-effective method, particularly for identifying the most common *Candida* strains [33]. In this study, comparison of sequencing and MALDI-TOF MS revealed species-level misidentifications for *C. albicans*, *C. parapsilosis*, *C. glabrata*, *C. krusei*, and *C. lusitaniae*, whereas *C. tropicalis*, *C. kefyr*, *C. rugosa*, and *C. fabianii* were identified with 100% accuracy by MALDI-TOF MS.

Accurate and rapid species identification is critical for guiding appropriate antifungal therapy. In this study, ITS rDNA sequencing was used as the reference method, enabling definitive species-level identification. When compared with sequencing results, MALDI-TOF MS demonstrated higher concordance than CHROMagar Candida, particularly for NAC species. While CHROMagar Candida remains a practical and cost-effective screening tool, its limited ability to reliably differentiate certain species—especially among NAC—was evident. In contrast, MALDI-TOF MS provided faster and more accurate identification across a broad range of species. Nevertheless, the implementation of MALDI-TOF MS may be constrained in resource-limited laboratories due to initial investment costs, and chromogenic media may still serve as a useful preliminary approach in such settings.

Virulence factors are essential for *Candida* pathogenicity by promoting colonization, tissue invasion, immune evasion, and persistence within the host [36].

Protease production contributes significantly to immune evasion by degrading host proteins such as albumin and α-macroglobulin [37]. Clinical studies have shown that protease activity is higher in *C. albicans* than in NAC species, although *C. tropicalis* consistently exhibits high protease activity among NAC species [38,39]. In this study, protease activity was detected in 70% of *C. albicans* isolates and in 40% of NAC isolates. Among the NAC species, the highest protease activity was observed in *C. krusei* (100%), while the lowest protease activity was seen in *C. rugosa* (0%).

Phospholipase enzymes contribute to *Candida* pathogenicity by promoting tissue invasion through the degradation of host cell membrane phospholipids, facilitating hyphal or pseudohyphal entry into host cells [40]. Clinical studies indicate that phospholipase activity is consistently higher in *C. albicans* than in NAC species, although the dominant NAC species showing the highest activity varies between studies [38,39]. While *C. tropicalis* and *C. glabrata* have been reported as the leading phospholipase-producing NAC species in some studies, others have identified *C. dubliniensis* as the most active, highlighting interspecies and regional variability [38,39,41]. In this study, 95% of *C. albicans* isolates and 6.6% of NAC isolates showed phospholipase activity. Among NAC species, no phospholipase activity was observed in the *C. tropicalis*, *C. krusei*, *C. kefyr*, and *C. rugosa* isolates.

In this study, protease and phospholipase activities were significantly more frequent in *C. albicans* than in NAC species, supporting previous reports that highlight the enhanced enzymatic arsenal of *C. albicans*. These hydrolytic enzymes contribute to host tissue damage and nutrient acquisition and may partially explain the continued clinical prominence of *C. albicans* despite the rising prevalence of NAC species.

Hemolysin production is an important virulence factor in *Candida* species, enabling blood cell lysis and iron acquisition from erythrocytes to support infection and growth. Nouraei et al. [41] observed the presence of hemolytic activity in all *C. albicans* strains that they are isolated, while in NAC species, hemolytic activity was found to be 91.83%. Furthermore, hemolytic activity has been documented in all *C. dubliniensis* and *C. krusei* strains. In this study, among the *Candida* strains included in the study, 55 (55%) showed two overlapping hemolysis zones (α + β hemolysis), 21 (21%) showed a single green zone (α hemolysis), while 24 (24%) showed no hemolytic activity. Marked differences were observed among *Candida* species, and the predominance of strong hemolysis in *C. albicans* and *C. tropicalis* indicates an enhanced iron acquisition capacity that may improve survival in iron-limited host environments.

*Candida* species exhibit different adhesion profiles. In a study, it was found that all *C. albicans* isolates (100%) showed weak adhesion, while *C. tropicalis* (37.5%) and *C. parapsilosis* (25.5%) isolates showed strong adhesion [42]. In this study, similar to literature, 43% of *Candida* isolates exhibited weak adhesion, with the highest rate being among *C. albicans* isolates (70%). Among the NAC species, it was observed that *C. tropicalis*, *C. krusei*, *C. rugosa*, and *C. lusitaniae* isolates did not show weak adhesion.

*Candida* species form biofilms on medical devices and mucosal surfaces via hyphae and pseudohyphae, contributing to persistent infections [43]. Several studies report variable biofilm formation rates among species, with NAC species often demonstrating equal or greater biofilm-forming capacity than *C. albicans*, although the dominant biofilm-producing species differs between studies [18,44,45]. Regional studies, particularly from India, further highlight substantial variability in biofilm production between *C. albicans* and NAC species, indicating species- and population-dependent differences [46]. In this study, biofilm formation was observed in nearly half of the isolates, with strong biofilm production most frequently associated with *C. parapsilosis*. This finding aligns with previous studies reporting a high biofilm-forming capacity for *C. parapsilosis*, particularly in catheter-related infections.

Retrospective and multicentre studies indicate variable antifungal resistance among *Candida* species, with fluconazole resistance generally ranging from low to moderate but remaining a major concern, particularly for *C. albicans* and NAC species [22,47,48]. Azole resistance, especially to fluconazole and itraconazole, is more frequently observed in *C. glabrata*, *C. tropicalis*, and *C. parapsilosis*, while *C. krusei* demonstrates intrinsic fluconazole resistance [15,48,49,50]. Regional studies reveal substantial geographic variability in resistance patterns, including notably high fluconazole resistance rates in some cohorts of *C. albicans* isolates [51,52]. Amphotericin B has consistently shown high efficacy with low resistance rates across most studies, although occasional intermediate susceptibility or resistance has been reported [47,48,49]. In our study, antifungal susceptibility testing revealed no statistically significant interspecies differences in MIC distributions for amphotericin B, fluconazole, itraconazole, or nystatin. Nevertheless, species-specific trends were observed. *C. albicans* isolates tended to exhibit higher fluconazole MIC values, whereas *C. tropicalis* generally displayed lower MICs across the tested agents. Such trends, although not statistically significant, may have clinical relevance, particularly in settings where empirical antifungal therapy is initiated before species identification is completed. It should be noted that differences between EUCAST and CLSI methodologies and breakpoint definitions may influence reported resistance rates and should be considered when comparing antifungal susceptibility data across studies.

Importantly, no significant associations were identified between individual virulence factors and antifungal MIC values. This finding supports the concept that antifungal resistance in *Candida* species is primarily driven by strain-level genetic mechanisms—such as efflux pump overexpression, target enzyme alterations, and stress-response pathways—rather than by isolated phenotypic virulence traits. Biofilm formation remains a major clinical concern due to its association with persistent infections and reduced antifungal susceptibility. While biofilm formation demonstrated a non-significant trend toward increased fluconazole resistance risk, larger studies incorporating molecular resistance profiling are required to clarify this relationship.

Several limitations of this study should be acknowledged. First, the single-center design may limit the generalizability of the findings. Second, rare *Candida* species were excluded from statistical analyses due to limited sample sizes, which may restrict interpretation of species diversity and resistance patterns. Third, echinocandins—currently recommended as first-line agents for invasive candidiasis—were not included in the susceptibility testing panel. This represents an important limitation and should be addressed in future studies. Finally, clinical outcome data was not available, precluding direct correlations between virulence traits, antifungal susceptibility, and patient prognosis.

Despite these limitations, the strengths of this study include the use of ITS sequencing for definitive species identification, comprehensive phenotypic characterization of virulence factors, and antifungal susceptibility testing performed according to current EUCAST guidelines. Collectively, these findings contribute to a better understanding of the epidemiology, pathogenic potential, and antifungal susceptibility profiles of *Candida* species in ICU settings and may support the optimization of diagnostic and therapeutic strategies.

## 4. Materials and Methods

### 4.1. Collection of Candida Isolates

This prospective study was conducted from July 2022 to March 2023 at Necmettin Erbakan University Medical Faculty Hospital. The study was approved by the KTO Karatay University Non-Drug and Medical Device Research Ethics Committee (Approval No: 41901325-200-36426, Date: 17 June 2022). Patients were included if they had been hospitalized in the ICU for over 48 h, had clinical signs of infection, and yielded at least one clinical specimen (urine, blood, catheter, abscess, or wound) from which a *Candida* species grew. Non-blood specimens were inoculated onto sheep blood agar and Eosin Methylene Blue (EMB) agar plates. Blood specimens were inoculated into blood culture bottles (Becton Dickinson, Franklin Lakes, NJ, USA) and incubated at 37 °C for 48–72 h. After incubation, suspected yeast colonies were identified using the VITEK 2 Compact^®^ system (bioMérieux, Marcy l’Etoile, France). Specimens identified as *Candida* spp. were stored in bead storage tubes and sent to the Biology Department of Gaziantep University for further analysis.

### 4.2. Candida Chromogenic Agar Medium

Stock *Candida* isolates stored in bead tubes were first subcultured on Sabouraud dextrose agar (SDA) to ensure purity. Pure cultures were then inoculated onto Candida Chromogenic Agar (Condolab Candida Chromogenic Agar, Madrid, Spain) plates. Plates were incubated at 37 °C under ambient atmosphere for 48 h. After incubation, colonies were identified by color and morphology: green-smooth colonies were interpreted as *C. albicans*, blue-smooth as *C. tropicalis*, cream-smooth as *C. parapsilosis*, lilac-smooth as *C. glabrata*, and purple-rough as *C. krusei* [53].

### 4.3. MALDI-TOF MS Analysis

Colonies of each *Candida* spp. grown on SDA for 48 h were sampled and spotted onto a 96-spot stainless steel target plate. For protein extraction, each spot was overlaid with 1 μL of 70% formic acid and allowed to dry for 5 min. The dried spots were then covered with 1 μL of the CHCA matrix solution (α-cyano-4-hydroxycinnamic acid) and air-dried. The prepared plate was loaded into a MALDI-TOF MS system (Bruker Daltonics, Bremen, Germany). Spectral data were analyzed using the Bruker Biotyper 3.1 software (library version 4613). The resulting peak spectra were compared against the reference database to identify organisms at the species, genus, or family level [34]. Agreement between MALDI-TOF MS, CHROMagar Candida, and ITS rDNA sequencing results was evaluated using percentage concordance. Although Cohen’s kappa statistics were not calculated, percentage concordance was considered appropriate given the categorical nature of species identification and the use of ITS rDNA sequencing as the definitive reference standard. ITS sequencing was considered the reference standard for species-level identification.

### 4.4. PCR and Sequencing

Genomic DNA was extracted from each isolate using a High Pure PCR Template Kit (Roche Diagnostics, Indianapolis, IN, USA). The ITS1-5.8S-ITS2 region of the rDNA (approximately 500 bp) was amplified by PCR using ITS1 (forward: 5′-TCCGTAGGTGAACCTGCGG-3′) and ITS4 (reverse: 5′-TCCTCCGCTTATTGATATGC-3′) primers (Sentebio, Ankara, Türkiye) as previously described. PCR was performed for 35 cycles (ramp rate 1.6 °C/s) following initial denaturation at 95 °C for 5 min. Each cycle consisted of 30 s at 95 °C, 45 s at 57.3 °C, and 1 min at 72 °C, with a final extension at 72 °C for 5 min. PCR products were electrophoresed on a 1.5% agarose gel at 100 V for 30 min [54] and then purified using the ExoSAP-IT kit. Sequencing was performed on an ABI Prism 310 Genetic Analyzer (Applied Biosystems, Foster City, CA, USA). The ITS sequences obtained were compared to the NCBI BLAST database to determine species [55].

### 4.5. Virulence Factor and Antifungal Activity Assays

Microscopic morphology of isolates was examined after crystal violet staining. Protease activity was determined on agar plates containing 1% bovine serum albumin (BSA) as described by Staib [56]. After incubation, the protease activity (PR_zo_) was calculated as the ratio of the diameter of the clear zone around a colony to the colony diameter. Results were interpreted as: PR_zo_ = 1 (negative, –); 0.64 < PR_zo_ < 0.99 (moderately positive, +); and PR_zo_ ≤ 0.64 (strongly positive, ++) [57].

Phospholipase activity was assessed on egg-yolk agar as described by Price et al. [58]. After incubation, phospholipase activity (PF_zo_) was similarly calculated and interpreted as: PF_zo_ = 1 (negative, –); 0.64 < PF_zo_ < 0.99 (moderately positive, +); and PF_zo_ ≤ 0.64 (strongly positive, ++) [57]. The PRzo and PFzo cutoff values used for the interpretation of protease and phospholipase activity were based on previously published methodologies. These thresholds have been validated in earlier studies using reference *Candida* strains.

Hemolytic activity was tested on 5% sheep blood agar using the method of Luo et al. [59]. After incubation, isolates were categorized by hemolysis pattern: no zone (γ-hemolysis), a green zone (α-hemolysis), a clear zone (β-hemolysis), or a double zone (α + β hemolysis, with inner clear and outer green zones).

Adhesion was evaluated using a 24-well plate assay. Wells were inoculated with a 1 McFarland suspension of *Candida* cells and incubated for 2 h. Wells were then washed with PBS, and adherent cells were stained with crystal violet. Under light microscopy, cell counts were performed in a 0.1 mm^2^ area and converted to cells/mm^2^. Adhesion scores were assigned as: 0 < cells ≤ 500 (weak, +); 501–999 (moderate, ++); and ≥1000 (strong, +++) [60].

Biofilm formation was measured by a microtiter plate assay [61]. After staining and washing, absorbance at 570 nm was recorded. A cutoff OD (OD_C) was calculated as the mean OD of negative controls plus three standard deviations. Isolates with OD ≤ OD_C were scored as biofilm-negative (–). If OD_C < OD ≤ 2 × OD_C, biofilm was considered weak (+); if 2 × OD_C < OD ≤ 4 × OD_C, moderate (++); and if OD > 4 × OD_C, strong (+++) [62].

A chi-square test was conducted and a heat map created to determine the distribution of virulence characteristics among species.

To determine the antifungal minimum inhibitory concentration (MIC) of *Candida* isolates, MIC to Amphotericin B, Itraconazole, Fluconazole, and Nystatin was assessed according to the EUCAST liquid microdilution method and evaluated according to the EUCAST E.Def 7.4 October 2023 guidelines [63]. As the MIC distributions were highly varied, a nonparametric test, the Kruskal–Wallis test, was used to perform the statistics and a box plot was drawn.

One hundred clinical *Candida* isolates were identified by ITS rDNA sequencing. Nine species were detected; their numbers and statistical significance were determined. For the statistical analysis, species with *n* ≥ 10 isolates (*C. albicans*, *C. parapsilosis*, *C. glabrata*, *C. tropicalis*) were included in the study. Species represented by fewer than ten isolates were excluded from comparative statistical analyses due to insufficient statistical power and unreliable inference.

The association between antifungal resistance and virulence factors was assessed using Spearman correlation, Mann–Whitney U testing, and binary logistic regression.

## 5. Conclusions

*Candida* species remain a major cause of healthcare-associated infections in intensive care units, with non-albicans *Candida* (NAC) species increasingly contributing to morbidity and mortality. In this study, *C. albicans*, *C. parapsilosis*, and *C. glabrata* were the most frequently isolated species, reflecting the evolving epidemiology of candidiasis in critically ill patients. MALDI-TOF MS demonstrated superior accuracy for species-level identification compared with chromogenic media, particularly for NAC species, while ITS rDNA sequencing remained the definitive reference method. Phenotypic analyses revealed significant interspecies differences in virulence-associated enzymatic activities, with *C. albicans* exhibiting higher phospholipase, protease, and hemolytic activities than NAC species. In contrast, adhesion and biofilm formation were broadly conserved across species, underscoring their fundamental role in *Candida* pathogenicity. No statistically significant associations were observed between virulence traits and antifungal susceptibility profiles, supporting the concept that antifungal resistance is primarily driven by strain-level genetic mechanisms—such as efflux pump overexpression, *ERG11* mutations, and biofilm-associated tolerance—rather than isolated phenotypic characteristics. Although biofilm formation showed a directional increase in fluconazole resistance risk, this trend did not reach statistical significance, highlighting the need for larger cohorts and genomic correlation studies.

From a clinical perspective, these findings emphasize the importance of combining accurate species-level identification with routine antifungal susceptibility testing to guide effective empirical and targeted therapy in intensive care units. The integration of rapid diagnostic tools such as MALDI-TOF MS with standardized susceptibility testing may support timely therapeutic decision-making and antifungal stewardship. Despite the strengths of this study—including definitive species identification by ITS sequencing, comprehensive phenotypic virulence assessment, and antifungal susceptibility testing performed according to EUCAST guidelines—certain limitations should be acknowledged. These include limited sample sizes for some species, right-censoring of fluconazole MIC values, and the absence of molecular resistance profiling, particularly echinocandin susceptibility data. Future large-scale, multicenter studies incorporating echinocandin susceptibility testing, molecular resistance mechanisms, and detailed clinical outcome data are warranted to better elucidate the complex interplay between virulence, antifungal resistance, and patient prognosis, and to support the development of optimized therapeutic strategies in the face of rising antifungal resistance.

## Figures and Tables

**Table 1:** Identification of *Candida* species based on CCA, MALDI-TOF and ITS sequence regions.

*Candida* Species	Sequence	MALDI-TOF MS		CCA	
	Number of Species	Specificity	Misidentified	Specificity	Misidentified
*Candida albicans*	39	34 (%87.8)	*C. glabrata* (%7.70) *C. parapsilosis* (%2.56) *C. tropicalis* (%2.56)	31 (%79.48)	*C. tropicalis* (%12.82) *C. parapsilosis* (%5.12) *C. glabrata* (%2.56)
*Candida parapsilosis*	22	18 (%81.82)	*C. albicans* (%9.09) *C. glabrata* (%9.09)	18 (%81.81)	*C. albicans* (%4.55) *C. glabrata* (%4.55) *C. tropicalis* (%9.09)
*Candida glabrata*	18	16 (%88.90)	*C. albicans* (%5.55) *C. parapsilosis* (%5.55)	12 (%66.67)	*C. parapsilosis* (%33.33)
*Candida tropicalis*	10	10 (%100)	-	8 (%80)	*C. albicans* (%10) *C. glabrata* (%10)
*Candida kefyr* (*Kluvyeromyces marxianus*)	3	3 (%100)	*-*	-	*C. glabrata* (%100)
*Candida krusei* (*Pichia kudriavzevii*)	2	2 (%100)	*-*	2 (%100)	*-*
*Candida lusitaniae* (*Clavispora lusitaniae*)	3	1 (%66.67)	*C. albicans* (%33.33)	-	*C. glabrata* (%100)
*Candida rugosa* (*Diutina rugosa*)	2	2 (%100)	*-*	-	*C. parapsilosis* (%50) *C. albicans* (%50)
*Candida fabianii* (*Cyberlindnera fabianii*)	1	1 (%100)	*-*	-	*C. parapsilosis* (%100)

MALDI-TOF MS correctly identified 100% of *C. tropicalis* (*n* = 10), *C. kefyr* (3), *C. rugosa* (2), and *C. fabianii* (1) isolates. Among *C. albicans* isolates (*n* = 39), MALDI-TOF MS misidentified 3 (7.70%) as *C. glabrata*, 1 (2.56%) as *C. tropicalis*, and 1 (2.56%) as *C. parapsilosis*. Among *C. parapsilosis* (*n* = 22), 2 (9.09%) were misidentified as *C. albicans* and 2 (9.09%) as *C. glabrata*. Among *C. glabrata* (*n* = 18), 1 (5.56%) was misidentified as *C. albicans* and 1 (5.56%) as *C. parapsilosis*.

**Table 2:** Species distribution, inclusion status, and justification.

Species	*n*	Inclusion Status	Justification
*Candida albicans*	39	Included	Adequate sample size (*n* ≥ 10)
*Candida parapsilosis*	22	Included	Adequate sample size (*n* ≥ 10)
*Candida glabrata*	18	Included	Adequate sample size (*n* ≥ 10)
*Candida tropicalis*	10	Included	Adequate sample size (*n* ≥ 10)
*Candida kefyr*	3	Excluded	*n* < 5, zero MIC variance
*Candida lusitaniae*	3	Excluded	*n* < 5, chi-square invalid
*Candida krusei*	2	Excluded	*n* < 5, regression separation
*Candida rugosa*	2	Excluded	*n* < 5, unreliable inference
*Candida fabianii*	1	Excluded	*n* < 5, insufficient variability

**Table 3:** Distribution of virulence traits among species (Chi-square test results).

Virulence Factor	χ^2^	*p*-Value	Interpretation
Protease	21.56	3.2 × 10^−5^	Highly significant
Phospholipase	64.40	<0.0001	Extremely significant
Hemolysis	11.10	0.011	Significant
Adhesion	0.00	1.00	Universal positivity
Biofilm	1.75	0.626	No significant difference

**Table 4:** Kruskal–Wallis comparison of MIC distributions across species (Kruskal–Wallis, *p* > 0.05).

Antifungal	H Statistic	*p*-Value	Interpretation
Amphotericin B	1.96	0.580	No significant difference
Fluconazole	2.89	0.408	No significant difference
Itraconazole	0.43	0.933	No significant difference
Nystatin	4.77	0.189	No significant difference

## Data Availability

The original contributions presented in this study are included in the article. Further inquiries can be directed to the corresponding author.
